# Impact of modified bladder neck suspension on early recovery of continence after robot-assisted radical prostatectomy (RARP)

**DOI:** 10.1007/s11701-023-01640-9

**Published:** 2023-06-19

**Authors:** Hyong Woo Moon, Seung Ah Rhew, Chang Eil Yoon, Hyeok Jae Kwon, Yong Hyun Park, Ji Youl Lee

**Affiliations:** Department of Urology, College of Medicine, Seoul St. Mary’s Hospital, The Catholic University of Korea, 222, Banpo-daero, Seocho-gu, Seoul, 06591 Republic of Korea

**Keywords:** Robot assisted radical prostatectomy, Incontinence, Functional outcome

## Abstract

The incontinence after RARP significantly decreases the quality of life in prostate cancer patients. A number of techniques have been introduced for the recovery of continence after RARP. Although, the mechanism of the continence recovery is still unclear. We aimed to evaluate the early recovery of continence after RARP by inducing early anterior adhesion and reducing the hypermobility of the urethra through the modified bladder neck suspension (BNS) procedure. From March 2018 to February 2020, a total of 227 consecutive patients who underwent RARP (by single surgeon) were included. Patients were divided into two groups based on operation procedure (Standard procedure vs BNS procedure). Demographics, perioperative variables, and pathologic outcome were analyzed. We assessed recovery of continence at 1, 3, 6 and 9 months after surgery. Postoperative recovery of continence defined as the use of no pad during 24 h. Multivariable logistic regression analyses were performed to evaluate independent predictors of the early recovery of continence at 1 month. We performed RARP with standard procedure (n = 106) or BNS procedure (n = 121). There was no statistical difference in perioperative variables between the two groups except anastomosis time (21.6 ± 12.9 vs 17.0 ± 7.6, *p* = 0.003). The pad free continence rate were 80.2% (standard group) and 91.3% (BNS group) at 9 month after RARP (*p* = 0.037). However, early continence rate (1mo) were significantly higher in the BNS group (12.3% vs 29.1%, *p* = 0.004). On multivariate logistic analyses, BNS procedure (odds ratio [OR] 2.78, 95% confidence interval [CI] 1.03–7.45, *p* = 0.0426), age (OR 0.92, CI 0.86–0.98, *p* = 0.0154) were independent factor for early recovery of continence after RARP. The modified bladder neck suspension procedure showed significantly better outcomes than the standard procedure in terms of the early recovery of urinary continence.

## Introduction

Robot-assisted radical prostatectomy (RARP) is being performed as the main treatment choice for localized prostate cancer. When compared to laparoscopic or retropubic radical prostatectomy, RARP does not show significantly favorable functional outcomes. However, RARP is currently one of the major treatment options, accounting for over 90% of radical prostatectomies performed in the United States [1–3]. Despite the understanding of pelvic structure and the introduction of various surgical techniques, postoperative incontinence remains one of the most important issues in patients undergoing radical prostatectomy (RP) [4]. Among the commonly used techniques, urologist perform RP to prevent incontinence using combinations of Rocco stitch, Patel stitch, anterior and posterior reconstruction, bladder neck preservation, and maximal urethral lengthening. Although the introduction of the Retzius-sparing technique has shown promising results in functional outcomes [5], this technique has limitations and cannot be used in all prostate cancer patients, including those with large prostates. Nerve sparing appears to be the most important aspect in radical prostatectomy. Comparing patients who underwent bilateral nerve sparing, unilateral nerve sparing, and those who did not undergo nerve sparing, relatively better outcomes in terms of continence and erectile function have been observed in the nerve sparing groups [6]. Furthermore, similar to Retzius-sparing, the nerve sparing technique may not be feasible in patients with locally advanced prostate cancer, where it may not be possible to perform bilateral nerve sparing.

Although the mechanism of continence recovery remains unclear, it is known that nerve sparing plays a crucial role in achieving favorable functional outcomes. However, in cases where Retzius-sparing and nerve sparing techniques cannot be utilized, anatomical support continues to be important. Therefore, we aimed to evaluate the early recovery of continence after RARP through the modified bladder neck suspension (BNS) procedure.

## Patients and methods

### Study population and design

From March 2018 to February 2020, a total of 227 consecutive patients who underwent RARP (by single surgeon) were included. Patients were divided into two groups based on operation procedure (Standard procedure vs BNS procedure). Demographics, perioperative variables, and pathologic outcome were analyzed. We assessed recovery of continence at 1, 3, 6 and 9 months after surgery. Postoperative recovery of continence defined as the use of no pad during 24 h. Informed consent was waived for all patients, and all data were collected retrospectively.

### Surgical technique

**Fig. 1:**
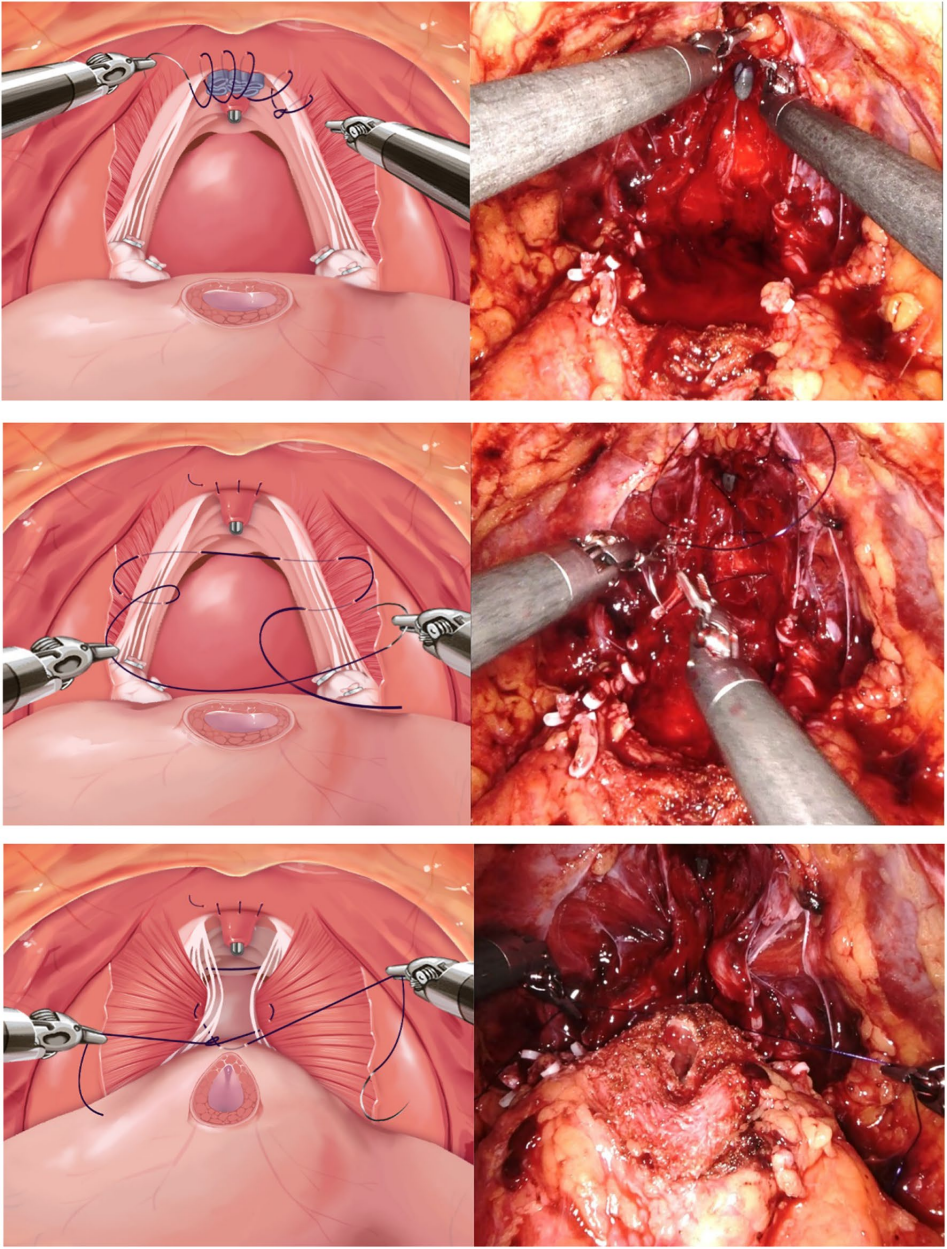

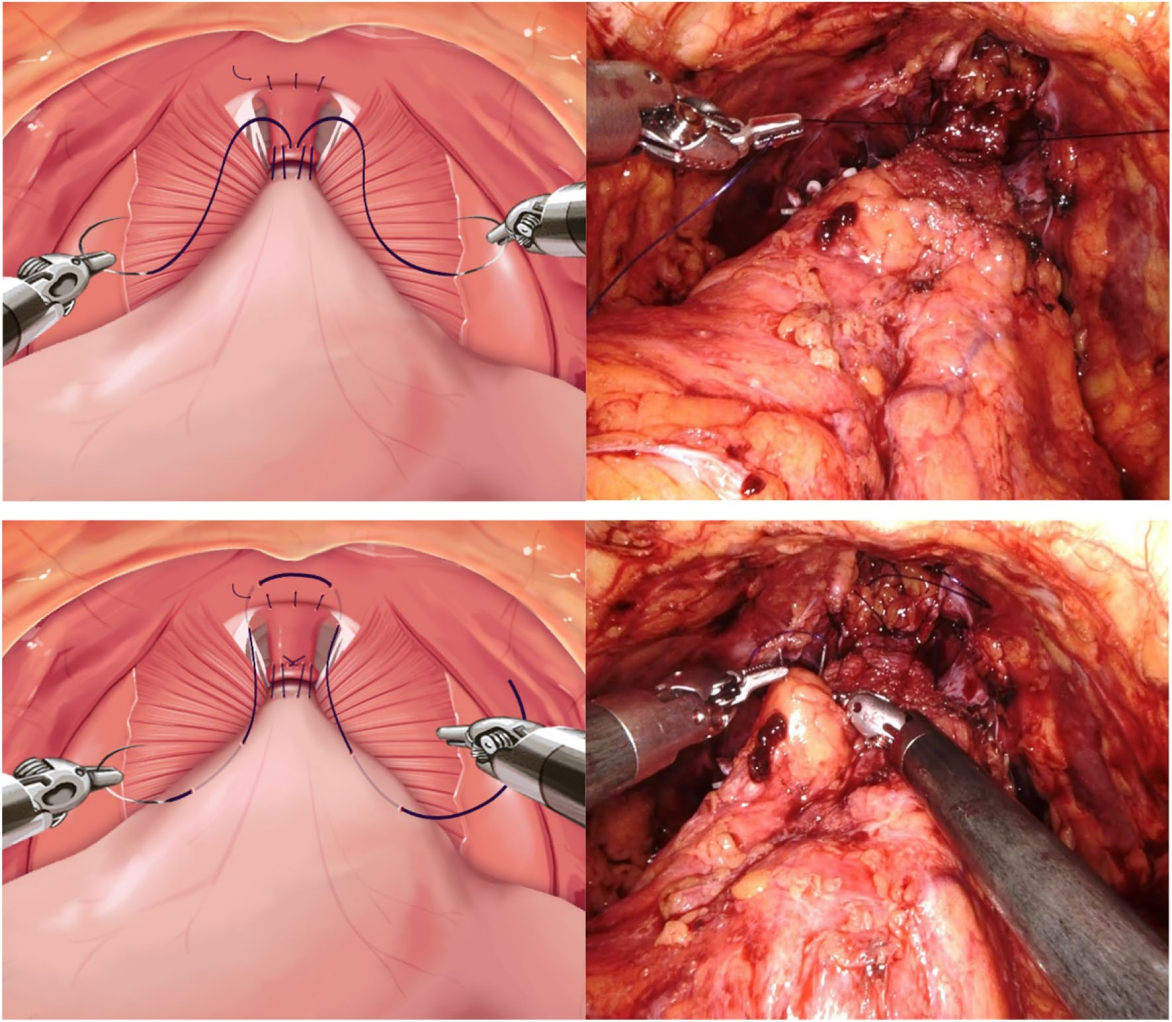
Schematic and operative view of modified bladder neck suspension

All patients underwent a transperitoneal approach, with the placement of four robot ports and two 11 mm assist ports into the peritoneal cavity. A perineotomy was performed to access the Retzius space and release the bladder. Further lateral dissection was carried out to identify and clip the vas deferens, followed by excision of periprostatic fat. Incision of the endopelvic fascia was performed to establish the boundaries of the prostate, with maximum preservation of the bladder neck. Bladder neck dissection was conducted, ensuring clear dissection between the bladder and prostate, and the seminal vesicles were dissected. After completing the dissection of the seminal vesicles, dissection was carried out in a posterolateral and apical direction along the Denonvilliers fascia of the prostate, with clipping of the bladder pedicle and nerve sparing performed according to individual patient requirements. Nerve sparing was performed in an antegrade fashion, with traction of the seminal vesicle using the third arm. After completing the apical dissection of the prostate, the dorsal vein complex was ligated using 3-0 V-Loc suture. Vesicourethral anastomosis was performed using a double-arm suture technique with 3–0 PDS, starting bidirectionally from the 6 o'clock position and followed by anterior repair. The schematic view and actual surgical photographs of these procedures are presented in Fig. 1.

In this study, all procedures were performed following this standard technique, and in the experimental group, additional bladder neck suspension was carried out before vesicourethral anastomosis, following complete removal of the prostate.

### Bladder neck suspension procedure

Bladder neck suspension is performed after the complete removal of the entire prostate and ligation of the dorsal vein complex. This procedure involves pulling the bilateral levator ani muscles to create a hammock-like structure. After pulling both levator ani muscles, and the bladder detrusor is tied with absorbable monofilament suture, facilitating the anastomosis between the urethra and bladder neck. Bladder neck suspension aims to provide mechanical support and improve continence outcomes by creating a supportive structure with the levator ani muscles. This technique helps stabilize the bladder neck and urethra.

### Data collection

The International Prostate Symptom Score (IPSS), Overactive Bladder Symptom Score (OAB-SS), International Index of Erectile Function (IIEF) and International Consultation on Incontinence Questionnaire (ICIQ) were used to evaluate functional and oncologic outcomes before and after surgery in both groups. Complications were recorded using the Clavien-Dindo classification. All surveys were compared at pre-surgery, 1 week after Foley catheter removal, 1 month, 3 months, and 6 months post-surgery. Continence was defined as a frequency of less than once a week and no need for pads.

### Statistical Analysis

Continuous variables were expressed as mean ± SD or range and were compared using independent samples t test and Mann–Whitney U test. Categorical variables were presented as numbers with percentages and compared using Chi-squared and Fisher’s exact test or using the linear-by-linear association test. We used a logistic regression model to do univariate and multivariate assessments to determine predictors of continence. p values < 0.05 were considered statistically significant. Statistical analysis was performed using R software (version 3.3.2, R Foundation for Statistical Computing, Vienna, Austria; http://r-project.org).

## Results

We performed RARP with standard procedure (n = 106) or BNS procedure (n = 121). There were no significant differences between the groups in terms of age (67.1 ± 6.8 years in the Standard group vs. 67.0 ± 6.7 years in the BNS group, p = 0.912), BMI (23.7 ± 3.1 kg/m^2^ vs. 24.5 ± 3.3 kg/m^2^, p = 0.054), ASA score (p = 0.514), TRUS volume (37.9 ± 15.7 cc vs. 35.1 ± 14.7 cc, p = 0.185), preoperative PSA levels (14.2 ± 22.3 ng/ml vs. 14.0 ± 22.0 ng/ml, p = 0.933), D’Amico risk (p = 0.658), preoperative IPSS (13.9 ± 8.3 vs. 11.8 ± 7.7, p = 0.187), and preoperative IIEF-5 scores (10.1 ± 7.1 vs. 10.5 ± 5.5, p = 0.891).

Console time was also similar between the groups (68.8 ± 25.3 min in the Standard group vs. 67.3 ± 21.4 min in the BNS group, p = 0.630), except for the anastomosis time, which was shorter in the BNS group (17.5 ± 7.3 min) compared to the Standard group (21.6 ± 12.9 min, p = 0.004). Blood loss (161.1 ± 124.7 mL vs. 135.0 ± 79.3 mL, p = 0.068) and prostate size (44.2 ± 14.0 g vs. 47.1 ± 17.0 g, p = 0.172) showed no significant differences.

Regarding nerve sparing, there was a trend towards higher bilateral nerve sparing in the BNS group (48.3%) compared to the Standard group (40.6%, p = 0.072). Clavien-Dindo complications did not significantly differ between the groups (p = 0.828). Pathologic staging (pT) and Gleason scores did not show significant differences either (p = 0.447 and p = 0.574, respectively). The rates of positive surgical margins (15.1% vs. 15.5%, p = 1.000) and pathologic node positivity (3.8% vs. 4.2%, p = 1.000) were similar between the groups.

Overall, the two groups were comparable in terms of demographic characteristics, surgical variables, pathologic findings, and complications, indicating a balanced comparison between the Standard and Bladder neck suspension groups (Table 1).

**Table 1:** Demographics in Standard group (n = 95) and Bladder neck suspension group (n = 91)

	Standard (N = 106)	BNS (N = 121)	p
Age(years)	67.1 ± 6.8	67.0 ± 6.7	0.912
BMI(kg/m^2^)	23.7 ± 3.1	24.5 ± 3.3	0.054
ASA score			0.514
2	87 (82.1%)	101 (84.2%)	
3	4 (3.8%)	7 (5.8%)	
TRUS volume (cc)	37.9 ± 15.7	35.1 ± 14.7	0.185
Preop PSA (ng/ml)	14.2 ± 22.3	14.0 ± 22.0	0.933
D’Amico risk			0.658
Low	8 (7.6%)	12 (10.0%)	
Intermediate	68 (64.8%)	71 (59.2%)	
High	29 (27.6%)	37 (30.8%)	
Preop IPSS	13.9 ± 8.3	11.8 ± 7.7	0.187
Preop IIEF-5	10.1 ± 7.1	10.5 ± 5.5	0.891
Console time (min)	68.8 ± 25.3	67.3 ± 21.4	0.630
Anastomosis	21.6 ± 12.9	17.5 ± 7.3	0.004
Blood loss(mL)	161.1 ± 124.7	135.0 ± 79.3	0.068
Prostate size(g)	44.2 ± 14.0	47.1 ± 17.0	0.172
Nerve sparing			0.072
Uni	12 (11.3%)	6 (5.0%)	
Bilateral	43 (40.6%)	58 (48.3%)	
Clavien-Dindo			0.828
2	2 (1.9%)	2 (2.5%)	
3	2 (1.9%)	1 (1.2%)	
pT			0.447
II	65 (61.3%)	78 (66.1%)	
IIIa	27 (25.5%)	25 (21.2%)	
IIIb	14 (13.2%)	13 (11.0%)	
IV	0 (0.0%)	2 (1.7%)	
Gleason Score total			0.574
6	8 (7.5%)	6 (5.1%)	
7	75 (70.8%)	82 (70.1%)	
8	14 (13.2%)	22 (18.8%)	
9	8 (7.5%)	7 (6.0%)	
10	1 (0.9%)	0 (0.0%)	
Positive surgical margin	16 (15.1%)	18 (15.5%)	1.000
Pathologic node positive	4 (3.8%)	5 (4.2%)	1.000

Table 2 presents the results of the continence rate at 1 week, 1 month, 3 months, 6 months, and 9 months after RARP without the use of pads. At 1 week post-surgery, the continence rate without pads was 3.0% in the standard group compared to 10.3% in the BNS group, showing a statistically significant difference (p = 0.026). Similarly, at 1 month, 3 months, 6 months, and 9 months post-surgery, the continence rate without pads was consistently higher in the BNS group compared to the standard group, with statistically significant differences observed at 1 month (12.3% vs. 32.5%, p = 0.002), 3 months (47.2% vs. 67.5%, p = 0.018), 6 months (67.9% vs. 85.4%, p = 0.035), and 9 months (80.2% vs. 96.5%, p = 0.011).

**Table 2:** Results of continence rate at 1 week, 1, 3, 6, 9 months after RARP (no pad)

	Standard (N = 106)	BNS (N = 121)	p
Continence (no pad)			0.005
1 week	3 (3.0%)	12 (10.3%)	
1 month	10 (10.0%)	26 (22.2%)	
3 month	38 (38.0%)	41 (35.0%)	
6 month	22 (22.0%)	21 (17.9%)	
9 month	13 (13.0%)	13 (11.1%)	
1 week continence	3 (3.0%)	12 (10.3%)	0.026
1 month continence	13 (12.3%)	38 (32.5%)	0.002
3 month continence	50 (47.2%)	79 (67.5%)	0.018
6 month continence	72 (67.9%)	98 (85.4%)	0.035
9 month continence	85 (80.2%)	112 (96.5%)	0.011

These results indicate that the additional BNS procedure resulted in significantly improved continence rates at various postoperative time points, suggesting its potential benefit in enhancing urinary continence outcomes after RARP.

Table 3 presents the results of the univariate and multivariate logistic regression analyses conducted to examine factors associated with early continence recovery after RARP.

**Table 3:** Univariate and multivariate logistic regression of early continence recovery after RARP (1 month)

	Univariate analysis			Multivariate analysis		
	OR	95% CI	*p*	OR	95% CI	*p*
Modified bladder neck suspension	2.94	1.43–6.04	0.0033	2.78	1.03–7.45	0.0426
Age	0.93	0.88–0.98	0.0048	0.92	0.86–0.98	0.0154
Nerve sparing	1.48	1.00–2.20	0.0512			
Preoperative IPSS	1.03	0.97–1.08	0.3230	1.04	0.99–1.10	0.1330
BMI	0.87	0.77–0.98	0.0234			

In the univariate analysis, the modified bladder neck suspension showed a significant association with early continence recovery, with an odds ratio (OR) of 2.94 (95% confidence interval [CI] 1.43–6.04, p = 0.0033). Age also demonstrated a significant association, with an OR of 0.93 (95% CI 0.88–0.98, p = 0.0048). Nerve sparing, preoperative IPSS, and BMI did not reach statistical significance in the univariate analysis.

In the multivariate analysis, after adjusting for other factors, the modified bladder neck suspension remained significantly associated with early continence recovery, with an OR of 2.78 (95% CI 1.03–7.45, p = 0.0426). Age also maintained its significant association, with an OR of 0.92 (95% CI 0.86–0.98, p = 0.0154). Nerve sparing, preoperative IPSS, and BMI did not show significant associations in the multivariate analysis.

These results indicate that the modified bladder neck suspension and younger age are independent factors associated with early continence recovery after RARP. However, nerve sparing, preoperative IPSS, and BMI did not demonstrate statistically significant associations with early continence recovery in the multivariate analysis.

## Discussion

In this study, we aimed to analyze the effect of BNS in RARP. We focused on the mechanical support aspect by implementing an additional bladder neck suspension procedure. Functional outcomes in RARP are influenced by various factors, including the surgical approach, such as whether Retzius sparing and NS are performed. The Retzius sparing technique aims to preserve the Retzius space, which may contribute to reduced bladder and urethral hypermobility, potentially enhancing postoperative continence outcomes. NS during RARP is a critical consideration as it aims to preserve the neurovascular bundles responsible for erectile function and continence.

While there is strong evidence supporting the importance of nerve-sparing RP in preserving erectile function, there is controversy over whether the NS technique improves postoperative urinary continence. Studies comparing bilateral NS, unilateral NS, and non-NS RARP have not found a significant difference in continence rates at one year after surgery [7]. This suggests that the physical preservation of cavernosal nerves may not have a significant impact on overall return to continence. Additionally, comparisons between interfascial and intrafascial NS RP have shown no statistically significant differences in continence rates at 12 months [8]. Moreover, it has been found that postoperative erectile function is not a reliable predictor of urinary continence, suggesting that anatomical factors, rather than nerve innervation, primarily contribute to continence outcomes after RP [9].

While RARP has shown higher continence rates compared to retropubic radical prostatectomy and laparoscopic prostatectomy, the time to achieve early continence after surgery varies depending on surgical techniques [1, 10]. Retzius sparing RARP, which preserves the Retzius space, has been reported to yield a continence rate of approximately 70% within one month post-surgery, showing superior outcomes compared to the transperitoneal approach [11–13]. This technique is speculated to reduce bladder and urethral hypermobility by preserving the Retzius space.

Various additional procedures can impact functional outcomes in RARP. These include anterior and posterior reconstruction, which involve the restoration of anatomical support and reconstruction of the puboprostatic ligament [14]. Bladder neck preservation techniques aim to maintain the integrity and function of the bladder neck, and urethral lengthening procedures focus on optimizing urethral support and continence [15, 16].

Considering the collective findings of these previous studies, it can be concluded that both nerve sparing and mechanical/anatomical support play crucial roles in the functional outcomes of RP. While nerve sparing is important for overall functional outcomes, it appears that mechanical support is particularly critical for the early recovery of continence.

In our study, we focused on the mechanical support aspect by implementing an additional bladder neck suspension procedure. We applied concepts from stress incontinence in females, where hypermobility of the urethra is a contributing factor. Through the BNS procedure, we aimed to reduce hypermobility and provide anatomical support. Particularly, in terms of early recovery after RP, our BNS demonstrated consistently favorable outcomes compared to the standard procedure in the control group, throughout all time periods following Foley catheter removal. This finding confirms that the provision of anatomical support, achieved through bladder neck suspension, aids in continence recovery. Our results are consistent with previous studies that have shown the benefits of functional reconstruction in early recovery of urinary continence after radical prostatectomy. Porpiglia et al. [17] conducted a nonrandomized study employing total anatomical reconstruction and reported favorable continence rates following catheter removal. Jeong et al. [18], in a randomized trial, reported that posterior reconstruction resulted in a shorter time to achieve continence compared to no reconstruction.

We acknowledge some limitations of our study, including the small sample size, lack of a randomized controlled trial (RCT), and the absence of an analysis on erectile function. However, it is important to note that our study group consisted of patients with low IIEF scores prior to surgery. Additionally, apart from patient factors, the surgical team's skill and experience play a significant role in the outcomes of radical prostatectomy [19]. Although our study was conducted by a single surgeon, the procedures were performed by the same team under the same surgeon, allowing us to confirm that BNS contributed to the improvement of surgical outcomes.

Overall, the implementation of the BNS procedure in RARP has demonstrated its potential to enhance surgical outcomes by providing mechanical support and reducing hypermobility, as supported by our study findings.

## Conclusions

The modified bladder neck suspension procedure demonstrated significantly superior outcomes compared to the standard procedure in terms of early recovery of urinary continence. Particularly, in cases RP for patients with locally advanced prostate cancer where nerve sparing is challenging or when nerve injury is anticipated, this surgical technique can serve as a favorable alternative for overcoming limitations in functional outcomes.

## Data Availability

Not applicable.
